# The use of ultrasonography in the diagnosis and management of Acute Kidney Injury and other forms renal dysfunction in the paediatric population: Systematic review protocol

**DOI:** 10.1371/journal.pone.0325549

**Published:** 2025-06-18

**Authors:** Gontse Leballo, Nabeela Arbee-Kalidas, Moses Mogakolodi Kebalepile, Palesa Motshabi Chakane

**Affiliations:** Department of Anaesthesiology, Charlotte Maxeke Johannesburg Academic, Hospital, University of the Witwatersrand, Johannesburg, South Africa; Universitas Pelita Harapan, INDONESIA

## Abstract

**Background:**

Acute kidney injury remains one of the most prevalent complications in hospitalised patients, with an especially high incidence in the paediatric population. Its diverse pathophysiological aetiologies present significant challenges in its clinical diagnosis. Relying solely on serum creatinine levels and urine output measurements for diagnosis has been shown to be inadequate, particularly for early detection of the disease. The wide range of disease manifestations highlights the importance of exploring diagnostic techniques that can identify patients at risk before disease progression occurs, thereby allowing for the implementation of timely corrective measures. Point-of-care ultrasound is a valuable technique with the potential to aid in the early identification of at-risk patients.

**Objective:**

To describe ultrasound parameters associated with the development of acute kidney injury or renal dysfunction in the hospitalised paediatric patient, and to examine their association with its development. This will include an analysis of the specific ultrasound features that may predict or be associated with the occurrence of acute kidney injury, as well as their potential role in identifying at-risk paediatric patients, facilitating timely interventions to mitigate further kidney damage.

**Methods:**

A systematic review of existing literature will be performed to assess ultrasound modalities currently associated with the diagnosis of acute kidney injury in the paediatric population. The protocol for this review has been registered with PROSPERO (CDR42024547614) and was developed in accordance with the guidelines set forth by the Preferred Reporting Items for Systematic Reviews and Meta-Analyses Protocols (PRISMA). A comprehensive search of scientific literature will be conducted across PubMed, Scopus, Cochrane Library, and CINAHL databases. The data search will be performed independently by the primary researchers (GL and NA), with any disagreements resolved by the third and fourth researchers (MK, PMC). The COVIDENCE Systematic Review Software® 2024 will be utilised for article exportation, review, and data extraction. The QUADAS-2 tool will be applied to assess the risk of bias in the diagnostic accuracy of the studies included in the review.

**Conclusion:**

This protocol outlines a systematic review designed to evaluate the application of ultrasonography in identifying paediatric patients at risk of developing acute kidney injury and other forms of renal dysfunction. The review will critically assess the effectiveness of ultrasound techniques, including point-of-care ultrasound, in identifying acute kidney injury in children, with a particular emphasis on early detection. The findings from this review will contribute to a deeper understanding of the role of ultrasonography in clinical practice, with the potential to improve patient outcomes through earlier intervention. The ultrasound techniques identified, including Doppler imaging and flow measurements, will be employed in a subsequent prospective study within the paediatric population to evaluate their role, sensitivity, and accuracy.

## Introduction

**Diagram 1:**
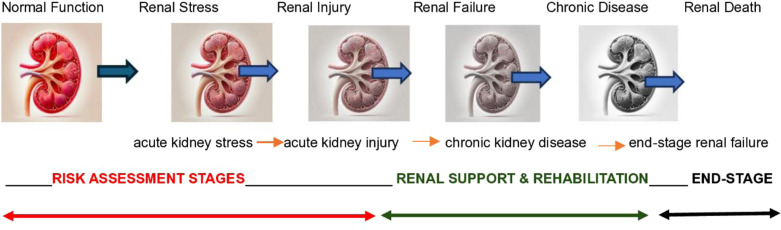
The spectrum of kidney function.

Acute kidney injury (AKI) is a multifactorial pathophysiological condition characterised by a rapid decline in renal function [1], typically subsequent to an episode of renal insult or stress, often referred to as acute kidney stress (AKS) [2]. In the absence of timely intervention, AKI may progress to renal failure, which can subsequently evolve into chronic kidney disease (CKD) and, in advanced stages, end-stage renal disease (ESRD), as illustrated in Diagram 1 below [3].

AKS is described as the “pre-injury” phase of renal dysfunction [2]. It is identified using novel biomarkers such as neutrophil gelatinase-associated lipocalin (NGAL), a proximal tubule biomarker [4], and cell-cycle arrest biomarkers, such as insulin-like growth factor binding protein (IGFBP-7) and tissue inhibitor of metalloproteinases (TIMP-2) [5]. Elevated urinary levels of NGAL within two hours following cardiopulmonary bypass (CPB) have been directly associated with the severity and duration of AKI [6].

AKI exhibits a high prevalence in hospitalised paediatric patients, particularly among those receiving care in an intensive care unit (ICU). The global incidence of AKI in paediatric patients is estimated to be 21% [7], with 27% of cases occurring in ICUs in low-to-middle income countries (LMICs), and 5% of cases involving non-critically ill paediatric patients [8,9]. One of the key predictors AKI in this population is age, with neonates, infants, and children representing 64%, 52%, and 9.6% to 42% of cases, respectively, and with children less than the age of two-years shown to be at highest risk [10]. This complication is associated with high morbidity and mortality rates [10–13], particularly following cardiac surgery involving the use of CPB [14,15]. In LMICs, the burden of AKI in the paediatric population is substantial, with high morbidity rates often linked to prolonged hospital stays [16,17]. Mortality rates are reported as high as 34%, significantly exceeding the global average of 14% [18]. These high prevalence rates may largely be attributed to disparities in diagnostic tools, limited access to healthcare, and the financial constrains faced by both populations and state healthcare facilities [19].

Validated AKI diagnostic and classification criteria are based on a rise in serum creatinine (SCr) levels and decreasing urine output measurements despite their shortcomings in identifying early kidney injury. A major disadvantage of SCr in AKI diagnosis is the delay in its increase which may take 24–48 hours from the initial renal insult [20]. Its detectable elevations occur only after approximately 50% of renal function has been lost [21]. Changes in SCr levels and urine output measurements consistently lag behind the onset of renal injury, making them ineffective for the early recognition of AKI [22]. In the paediatric population, the Kidney Disease Improving Global Outcomes (KDIGO) criteria and the paediatric Risk Injury Failure Loss and End-Stage (pRIFLE) classification are often employed, with the Acute Kidney Injury Network (AKIN) classification mentioned rarely in some literature [10,23]. The modified pRIFLE criteria utilises glomerular filtration rate (GFR) criteria, which is based on SCr and/or urine output measurements in classifying AKI into different stages [24]. SCr is used to calculate for estimated creatinine clearance (eCCr) by means of the Schwartz formula [10,25].

The multifactorial nature of AKI is underpinned by a complex interplay of diverse pathophysiological mechanisms. The factors contributing to AKI can be broadly categorised into renal pathologies (such as renal obstruction and exposure to exogenous or endogenous toxins), systemic factors (including sepsis, volume depletion, ischemia-reperfusion injury, and heart failure), and surgical factors [26]. This pathophysiological interplay has been demonstrated in various studies, which indicate that alterations in renal blood flow patterns, along with changes in the renal cortex and medullary echogenicity, can be associated with the AKI onset and its progression [27–29].

Aspects of point-of-care ultrasound (POCUS) are emerging as valuable resources in the diagnosis and management of patients with AKI in ICU settings [30]. The Chinese Critical Ultrasound Study Group (CCUSG) developed the Critical Care Ultrasound Guided (CCUSG)-A_(AKI)_BCDE protocol, which provides a comprehensive framework for the risk assessment and diagnosis of AKI in critical care patients [26]. This protocol offers a systematic approach to renal function assessment by evaluating the clinical profile that may contribute to the development of AKI, utilising B-mode ultrasonography for renal structural assessment, employing colour and spectral Doppler imaging of the kidneys, and assessing microcirculation. The development of this protocol highlights the essential role of renal ultrasound in facilitating the diagnosis and risk stratification of AKI, particularly in resource-constrained environments.

Doppler ultrasound has demonstrated potential as a cost-effective, non-invasive, and a rapid modality for predicting AKI in critically ill paediatric patients by identifying altered renal blood flow patterns [21,31]. Doppler measurements of hepatic, portal, cardiac, and renal vascular parameters have been shown to be associated with AKI in adult patients [32–34]. A study by Guinot et al. demonstrated that right ventricular systolic dysfunction and elevated central venous pressure (CVP) following cardiac surgery were associated with an increased risk of AKI [37]. Similarly, Pettey et al. reported that abnormal hepatic vein flow patterns and ratios, were correlated with AKI in adults undergoing cardiac surgery, with elevated CVP measurements independently associated with AKI [32]. Additionally, tricuspid annular plane systolic excursion (TAPSE) was found to be significantly associated with AKI in critically ill patients [38].

Fluid overload and thus venous congestion leads to an increase in venous pressure, which causes a decrease in renal blood flow and contributes to the occurrence of AKI [35]. The venous excess ultrasound (VExUS) score associated renal venous congestion with AKI [35]. It systematically assesses inferior vena cava (IVC) diameter and Doppler waveforms of hepatic, portal, and intrarenal veins, classifying the severity into Grades I to III [35,36]. Studies on paediatric renal arterial ultrasound parameters are predominantly reporting renal resistive index (RRI), and renal pulsatility index (RPI), with inconclusive and poor results [24,41]. Structural abnormalities on ultrasound typically present late and are not useful in AKI [29,39,40].

Studies utilising POCUS in adults have provided valuable insights, associating renal, hepatic, and cardiac ultrasound parameters to the occurrence of AKI. These findings underscore the potential of ultrasound techniques to improve early detection and monitoring of AKI in high-risk patients, particularly in resource-limited settings, and highlight the need for further research in paediatric populations to optimise diagnostic approaches. This systematic review aims to evaluate renal, hepatic, portal and cardiac ultrasound parameters associated with the development of AKI in paediatric patients.

Recommendations by the Enhanced Recovery after Cardiac Surgery (ERAS-Cardiac) support efforts to explore robust and cost-effective methods of early identification of renal stress following cardiac surgery, particularly ultrasound techniques that can quantify renal congestion [41]; and to employ Goal Directed Fluid Therapy (GDFT) practises to achieve required haemodynamic goals thus promoting oxygen delivery and end-organ perfusion [41,42].

## Aim

The aim of this review protocol is to outline the process for investigating clinically relevant ultrasound parameters (B-mode, Doppler and waveform ultrasound patterns) associated with the development of AKI in hospitalised paediatric patients.

## Objectives

The objective of this systematic review is to describe ultrasound parameters associated with the development of AKI or renal dysfunction in the hospitalised paediatric patient.

## Methodology

### Study design

**Fig 1:**
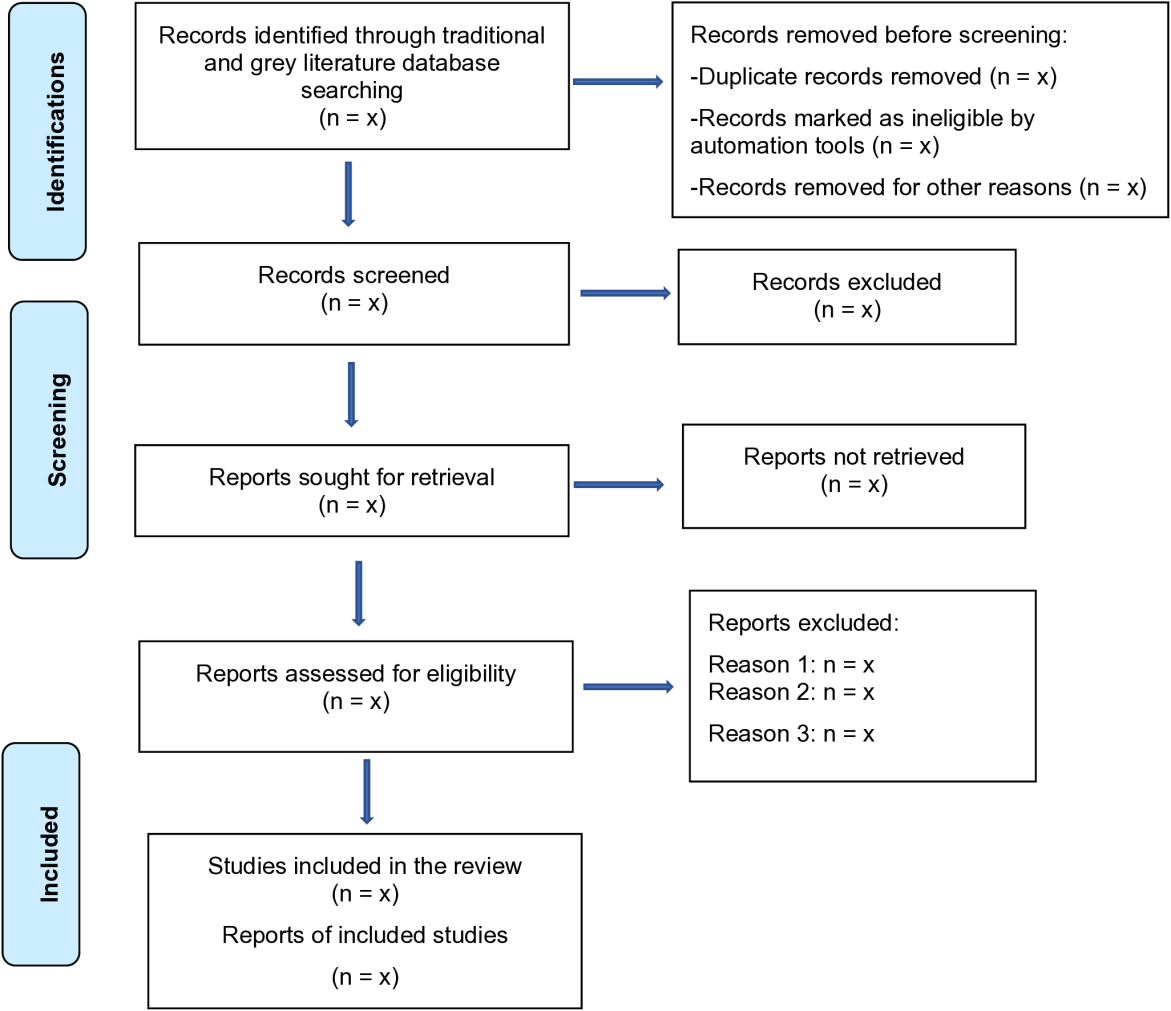
A PRISMA flow diagram showing a schematic representation of how literature screening will occur for the systematic review.

The protocol is developed according to the recommendations from the Preferred Reporting Items for Systematic Review and Meta-Analysis Protocols (PRISMA) guidelines (Fig 1) (PRISMA-P checklist- Appendix A, S1) [43,44]. It is registered on the International Prospective Register of Systematic reviews (PROSPERO 2024, CDR42024547614). While ethical clearance is not required for this review, the associated large-scale study has received approval from the Human Research Ethics Committee (HREC) Medical of the University of the Witwatersrand, South Africa (Reference number: M231003 M240423-A-0001).This protocol is designed to conduct a comprehensive systematic review of the literature on ultrasound modalities associated with the diagnosis of AKI or renal dysfunction in the paediatric population. All studies describing various ultrasound parameters such as renal structural findings, Doppler patterns, and waveform parameters explored clinically to identify paediatric patients with AKI will be included.

The following research assumptions are made: **paediatric patient:** a patient less than the age of 18 years; **acute kidney injury:** it will be inclusive of AKI, acute renal failure (ARF), renal injury, and renal dysfunction; **acute kidney injury diagnostic criteria:** in this study AKI will be defined according to the paediatric Risk, Injury, Failure, Loss, and End-Stage renal function (pRIFLE) criteria, Kidney Disease Improving Global Outcomes (KDIGO) criteria, and the Acute Kidney Injury (AKIN) network; and **ultrasound parameters:** they will include Doppler haemodynamic flow patterns (renal, hepatic, and cardiac), and renal morphological, or anatomic structural changes.

The study aims to identify and emphasise evidence-based bedside ultrasound parameters that are correlated with the diagnosis of AKI or renal dysfunction in paediatric patients. The study will follow a seven-step methodological framework based on the PRISMA guidelines [45].

**Step 1: Identifying the research question.** The population, exposure, and outcome (PEO) framework (Table 1) will be applied. The proposed systematic review will address the following research question:

**Table 1:** The population, exposure, and outcomes (PEO) framework.

Population	Paediatric patients (< 18 years of age)
Exposure of interest or Intervention	B-mode ultrasound, Doppler flow patterns and wavelength patterns (renal/ hepatic/ portal), right heart chamber measurements associated with AKI diagnosis
Comparator or Control	none
Outcomes	Acute kidney injury or renal dysfunction

Which bedside ultrasound techniques can clinicians utilise for risk assessment of AKI in hospitalised paediatric patients?

**Step 2: Eligibility criteria for considering studies for inclusion in this review.** The inclusion of the identified studies will be conducted independently by two primary reviewers (GL and NA), with the third (MK) and fourth reviewers (PMC) consulted in cases of discordance. The selected studies will be systematically reviewed by GL and NA, and the relevant data (Table 2) will be presented in alignment with the research question and the PEO framework (Table 1). Searches will be performed for English-language literature, along with unpublished and grey literature, spanning the past 40 years (1984–2024). The inclusion and exclusion criteria are detailed in Table 2. Only studies conducted in accordance with established methodological standards will be included.

**Table 2:** Inclusion and exclusion criteria for this systematic review.

	Inclusion criteria	Exclusion criteria
Population	Paediatric patients (age ≤ 18 years)	Duplicate studies
Study design	Cohort studies, case control studies, *RCTs, grey literature, master’s, and PhD Thesis	
Outcome	*AKI diagnosis (or any phrase utilised to describe renal dysfunction) utilising bedside ultrasound techniques or *POCUS	Non-English studies
Publication type	Full text in English	

*RCT: randomised control trials; *AKI: acute kidney injury; *POCUS: point-of-care-ultrasound.

**Step 3: Information sources and search strategy for the identification of relevant studies.** The literature search will encompass the following databases: MEDLINE (PubMed), Scopus, Cochrane Library, and CINAHL. In addition to the electronic database search, the reference lists of eligible articles will also be reviewed.

A search strategy for data collection from these databases is outlined in Table 3 below. Medical Subject Headings (MeSH) terms and relevant keywords such as “paediatric patient,” “ultrasound,” and “acute kidney injury” will be employed during the search.

**Table 3:** Databases search strategy according to the population, exposure, and outcomes (PEO) framework.

Database	PEO Framework	MeSH terms/ Phrases
**PubMed/ MEDLINE**	**Population**	((((((((((((pediatrics[MeSH Terms]) OR (“paediatric patient*”[Text Word])) OR (“pediatric patient*”[Text Word])) OR (“paediatric patient*”[Title/Abstract])) OR (“pediatric patient*”[Title/Abstract])) OR (child[Text Word])) OR (children[Text Word])) OR (neonate*[Text Word])) OR (child[Title/Abstract])) OR (children[Title/Abstract])) OR (neonate*[Title/Abstract])) OR (infant*[Title/Abstract])) OR (infant*[Text Word])
	**Exposure of interest**	((((acute kidney injury[MeSH Terms]) OR (“acute renal failure”[Text Word])) OR (“acute renal failure”[Title/Abstract])) OR (“acute kidney insufficiencies”[Text Word])) OR (“acute kidney insufficiencies”[Title/Abstract])
	**Outcomes**	“ultrasound”[Title/Abstract] OR “ultrasonograph*”[Title/Abstract] OR “POCUS”[Title/Abstract] OR “ultrasonogra*”[Title/Abstract] OR “echocardiogra*”[Title/Abstract] OR “echocardiograph*”[Title/Abstract] OR “doppler”[Title/Abstract] OR “portal ultrasound”[Title/Abstract] OR “renal ultrasound”[Title/Abstract] OR “hepatic ultrasound”[Title/Abstract] OR “cardiac ultrasound”[Title/Abstract] OR “resistive index”[Title/Abstract] OR “portal vein pulsatility index”[Title/Abstract] OR “portal vein velocity”[Title/Abstract] OR “hepatic vein Doppler”[Title/Abstract] OR “hepatic vein velocity”[Title/Abstract] OR “inferior vena cava diameter”[Title/Abstract] OR “ejection fraction”[Title/Abstract] OR “EF”[Title/Abstract] OR “LVEF”[Title/Abstract] OR “tricuspid annular plane systolic excursion”[Title/Abstract] OR “TAPSE”[Title/Abstract] OR “mitral annular plane systolic excursion”[Title/Abstract] OR “MAPSE”[Title/Abstract] OR “cardiac output”[Title/Abstract] OR “cardiac index”[Title/Abstract] OR “parenchymal echogenicity”[Title/Abstract] OR “RRI”[Title/Abstract] OR “renal circulation”[MeSH Terms] OR “renal blood flow, effective”[MeSH Terms] OR “renal ultrasound”[Text Word] OR “renal resistive index”[Text Word] OR “renal doppler”[Text Word]
**Scopus**	(TITLE-ABS-KEY (“acute kidney injury” OR “acute renal failure” OR “renal dysfunction” OR “AKI”) AND TITLE-ABS-KEY (“pediatric*” OR “paediatric*” OR “infant” OR “neonate”) AND TITLE-ABS-KEY (“ultrasound” OR “doppler” OR “ultrasonogra*” OR “renal ultrasound” OR “renal resistive index” OR “RRI” OR “hepatic ultrasound” OR “cardiac ultrasound” OR “FATE” OR “hepatic vein ratio”)) AND (LIMIT-TO (SUBJAREA, “MEDI”)) AND (LIMIT-TO (EXACTKEYWORD, “Human”) OR LIMIT-TO (EXACTKEYWORD, “Acute Kidney Failure”) OR LIMIT-TO (EXACTKEYWORD, “Infant, Newborn”) OR LIMIT-TO (EXACTKEYWORD, “Child”) OR LIMIT-TO (EXACTKEYWORD, “Newborn”) OR LIMIT-TO (EXACTKEYWORD, “Infant”) OR LIMIT-TO (EXACTKEYWORD, “Humans”)) AND (LIMIT-TO (LANGUAGE, “English”)) AND (EXCLUDE (DOCTYPE, “re”) OR EXCLUDE (DOCTYPE, “ch”))	
**Cochrane Library**	(Paediatrics AND Acute kidney injury OR AKI OR Acute renal failure OR acute renal dysfuntion):ti,ab,kw AND (“POCUS” OR “Renal resistive index” OR “RRI” OR “hepatic doppler” OR “hepatic vein ratio” OR “echocardiograph” OR “FATE”):ti,ab,kw NOT (adult):ti,ab,kw	
**CINHAL**	TI (acute kidney injury or acute renal failure or aki or acute kidney failure) AND AB (ultrasound or sonography or sonogram or ultrasonography) AND AB (paediatric or pediatric or children	

The results of the search will be managed using the EndNote™ 20 [Endnote 20.6 (2001–2020), Clarivate Analytics (US) LLC, Philadelphia, PA] reference management tool. Eligible studies will be exported to the Covidence [Covidence systematic review software (2024), Veritas Health Innovation, Melbourne, Australia] data extraction software for systematic data extraction.

**Step 4: Study selection and eligibility.** The study search and selection of eligible articles will follow a multistep process, independently conducted by primary reviewers GL and NA, adhering to the PRISMA guidelines as illustrated in Fig 1.

The screening process will be implemented as follows:

i **Title screening:** The aforementioned databases (PUBMED, Scopus, Cochrane Library, and CINHAL) will be utilised to identify eligible studies, with primary reviewers applying predefined inclusion and exclusion criteria during the screening process. The results from the database searches will be exported to EndNote™ 20 [Endnote 20.6 (2001–2020), Clarivate Analytics (US) LLC, Philadelphia, PA], and subsequently transferred to Covidence [Covidence systematic review software (2024), Veritas Health Innovation, Melbourne, Australia] for further study screening. Covidence will automatically remove duplicate entries. Both reviewers will review all study titles against the established eligibility criteria. Any disagreements regarding study inclusion during title screening will be resolved by the third and fourth reviewers (MK and PMC) prior to proceeding to the studies abstract screening.
ii **Abstract screening:** Upon completion of the title screening stage and resolution of any disagreements, the primary reviewers will independently screen the abstracts of the included studies in parallel within Covidence software. Studies whose titles and abstracts do not meet the predefined inclusion criteria will be excluded. Any discrepancies encountered by the primary reviewers during this process will be addressed and resolved by the third and fourth reviewers.
iii **Full article screening:** Full-text articles of studies included during the abstract screening phase will be imported into Covidence software for full-text screening. Similar to the previous stages (title and abstract screening), the primary reviewers will independently assess the full-text articles of the included studies. A thorough evaluation of each study will determine the inclusion of relevant studies and the exclusion of those that do not meet the eligibility criteria. Any discrepancies between the primary reviewers will be resolved by the third and fourth reviewers.

**Step 5: Data extraction**. Upon completion of the full-text article screening phase, primary reviewers (GL and AN) will independently extract data from all included studies. To minimise bias, inter-rater variability, and data extraction errors, the data extraction process will be performed separately by each reviewer using a pre-established data extraction template (Table 4), which was developed prior to the commencement of the screening process in Step 4. The extracted data from eligible articles will be verified by the third (MK) and fourth (PMC) reviewers and categorised as follows (as outlined in Table 4: Data Collection Table):

**Table 4:** Data collection template for the systematic review.

1. Study identification Sponsorship source: Country: Setting: Comments:
**2. Authors’ details** Name: Institution: Email address: Physical Address:
**3. Study period and other** Start date: End date: Single centre: Multicentre:
**4. Methods** Study design:
**5. Criteria** Inclusion: Exclusion:
**6. Population** Mean age (years) ‡SD: Medical/ non-surgical cases/ admissions: Surgical Cases/ admissions: Sample size:
**7. Intervention (ultrasound technique utilised for diagnosis)** Renal parameters (RRI/ RPI/other): Cardiac parameters (CVP/ IVC/ TAPSE/ RV-pressure/ other): Hepatic parameters (HVR/ other): Portal parameters (PVP index/ other):
**8. Outcome (acute renal dysfunction diagnosis: dichotomous outcome)** Acute kidney injury/ Acute renal failure/ Other:
**9. Serum Creatinine/ Urine Output/ Biomarker parameter also utilised** KDIGO: pRIFLE: AKIN: GFR: NGAL: Cystatin C: TIMP-2/ IGFBP-7: Other: None:
**10. Statistical analysis** - Continuous measures standard deviation (mean/ SD/ N): confidence intervals (mean/ CI/ N): - Dichotomous measures percentage of participants with event (N/ %): number of participants with event (n/ N)

i **General study details**: including author(s), year of publication, study title, country of study, and main conclusions.
ii **Participant characteristics**: such as sample size, patient selection criteria, gender, type of hospital admission (e.g., medical ICU or post-surgery ICU), and presence of AKI or renal dysfunction.
iii **Study methods**: including study design (e.g., cohort, observational, pilot, or RCT) and whether the study was single or multicentre.
iv **Ultrasound parameters employed**: renal Doppler ultrasound parameters, inclusion of hepatic or cardiac ultrasound, and renal structural measurements.
v **Standard AKI diagnostic criteria**: utilisation of SCr and/or urine output measurements as the standard diagnostic criterion, including KDIGO, pRIFLE, and AKIN criteria.
vi **Renal biomarkers:** data from studies that mention the use of renal biomarkers will also be extracted; their method of measurement, and their relevance to the diagnosis or monitoring of AKI or other forms of renal dysfunction. The data will be categorised according to the type of biomarkers (e.g., serum, urinary, or molecular markers) and any associated outcomes, such as diagnostic accuracy or prognostic value.
vii **Outcomes**: including AKI diagnosis, other forms of renal dysfunction, associated complications, and mortality rates.

**Step 6: Assessment of study quality and risk of bias.** Assessment of risk of bias of the included studies in this review paper will be performed using the Quality Assessment of comparative Diagnostic Accuracy Studies (QUADAS) −2 tool (Appendix B) [46]. It is a tool recommended for evaluation of risk of bias of diagnostic accuracy. The GRADE guidelines [47], will be used to evaluate publication bias and selective reporting of included studies and be presented as plots of outcome variables against sample size.

**Step 7: Data synthesis and analysis of results.** Extracted data addressing the objectives will be presented in tables. Where data allows, a meta-analysis of outcome will be conducted and reported in the form of mean differences for continuous outcomes, odds ratios for dichotomous data, 95% confidence intervals and two-sided p-values for each outcome. A p-value < 0.05 will be considered statistically significant. A narrative synthesis will provide evidence of the pooled prevalence, corresponding standard error and 95% confidence intervals of age, setting and acute AKI diagnosed using ultrasound parameters.

Should a meta-analysis be feasible, fixed effect and random effect models will be used to pool data from individual prevalence and incidence rate estimates from the studies. The heterogeneity of studies included in the meta-analysis will be assessed through the Cochran’s Q test (P value <0.05) and I^2^ (>50%). The inverse-variance weighting techniques will be used to adjust for publication bias due to study size.

The results will be presented in the form of summary tables and flowcharts, which will be included in the final manuscript. The outputs of this review will be disseminated through publications in peer-reviewed journals, conference presentations, and reports for relevant stakeholders.

### Differences between protocol and review

In accordance with the published protocol, we will conduct a review and document any discrepancies from the protocol. Any modifications will be reflected by updating the PROSPERO record and detailing these changes in a section titled “*Differences Between the Protocol and the Review*” in the published systematic review results article.

### Ethical considerations

This review article forms part of a broader research initiative investigating perioperative AKI in paediatric cardiac patients. The ultrasound parameters identified and validated through this systematic review will be employed in a large-scale prospective study aimed at identifying paediatric patients at risk for AKI. This will involve comparing the identified ultrasound parameters—specifically Doppler flow patterns, blood flow resistance ratios in the liver and kidneys, as well as right-sided cardiac pressures—with an established AKI diagnostic criterion (KDIGO), which is based on serum creatinine (SCr), urine output measurements, and renal molecular biomarkers (including serum Cystatin C, IGFBP-7, and TIMP-2).

### Study data

Raw data collected from all eligible studies will be exported to Microsoft Excel (Microsoft Corporation, 365). This data will be made publicly available without any restrictions and will be submitted as part of the Supporting Information Files upon submission of the completed study to the journal.

## Conclusion

This systematic review, with the potential for meta-analysis, aims to critically assess the use of ultrasound parameters that are associated with paediatric patients diagnosed with AKI based on established diagnostic criteria. The primary objective is to delineate the at-risk population for AKI and to identify specific ultrasound characteristics that effectively aid in its diagnosis. Furthermore, the review will examine the limitations associated with the application of these ultrasound modalities.

## Supporting information

S1 Appendix A PRISMA-P checklist 2015 checklist: recommended items to address in a systematic review protocol. (DOCX)
