# Supplementary material for: The use of ultrasonography in the diagnosis and management of Acute Kidney Injury and other forms renal dysfunction in the paediatric population: Systematic review protocol
Source: PLoS One. 2025 Jun 18;20(6):e0325549. doi: 10.1371/journal.pone.0325549 (PMC12176226; doi:10.1371/journal.pone.0325549)
Supplement: S1 Appendix A — (DOCX) [file pone.0325549.s001.docx]

**APPENDIX A: PRISMA-P Checklist**

**PRISMA-P (Preferred Reporting Items for Systematic review and Meta-Analysis Protocols) 2015 checklist: recommended items to address in a systematic review protocol***

| Section and topic | Item No | Checklist item | Submitted PROTOCOL details as per the PRISMA-P checklist |
| --- | --- | --- | --- |
| ADMINISTRATIVE INFORMATION | | | |
| **Title:** |  |  | **The use of ultrasonography in the diagnosis and management of acute kidney injury and other forms of renal dysfunction in the paediatric population: systematic review protocol**  **(Title page of the protocol – page 1 of the protocol manuscript)** |
| Identification | 1a | Identify the report as a protocol of a systematic review | **PROTOCOL for a Systematic Review** |
| Update | 1b | If the protocol is for an update of a previous systematic review, identify as such | **N/A** |
| Registration | 2 | If registered, provide the name of the registry (such as PROSPERO) and registration number | **Registered with PROSPERO: CDR42024547614**  **(Page 5: Methodology Section)** |
| **Authors:** |  |  |  |
| Contact | 3a | Provide name, institutional affiliation, e-mail address of all protocol authors; provide physical mailing address of corresponding author | ***Gontse Leballo ^1^, Nabeela Arbee-Kalidas ^1^ , Moses Kebalepile ^1^, Palesa Motshabi Chakane ^1^**  **^1^ Department of Anaesthesiology, Charlotte Maxeke Johannesburg Academic Hospital; University of the Witwatersrand, Johannesburg, South Africa**  Gontse Leballo, **ORCID**:[0000-0003-2217-6288](https://orcid.org/0000-0003-2217-6288)  Nabeela Arbee-Kalidas, **ORCID**:[0000-0002-1343-3636](https://orcid.org/0000-0002-1343-3636)  Moses Kebalepile, **ORCID**:[0000-0002-5346-5798](https://orcid.org/0000-0002-5346-5798)  Palesa Motshabi Chakane, **ORCID**:[0000-0001-9990-6336](https://orcid.org/0000-0001-9990-6336)  *Corresponding author: [gleballomothibi@gmail.com](mailto:gleballomothibi@gmail.com)  Gontse Leballo, Charlotte Maxeke Johannesburg Academic Hospital, Area 361, Department of Anaesthesiology, Parktown, Johannesburg, South Africa, 2193  Nabeela Arbee-Kalidas: [nabeela_arbee@yahoo.com](mailto:nabeela_arbee@yahoo.com)  Moses Kebalepile: [moses.kebalepile@wits.ac.za](mailto:moses.kebalepile@wits.ac.za)  Palesa Motshabi Chakane: [palesa.motshabi@wits.ac.za](mailto:palesa.motshabi@wits.ac.za) |
| Contributions | 3b | Describe contributions of protocol authors and identify the guarantor of the review | **Guarantor: Dr Gontse Leballo**  **Main Author: Dr Gontse Leballo**  **Assistant Author: Dr Nabeela Arbee-Kalidas**  **Assistant Author and Statistics: Dr Moses Kebalepile**  **Senior Author: Professor Palesa Motshabi Chakane** |
| Amendments | 4 | If the protocol represents an amendment of a previously completed or published protocol, identify as such and list changes; otherwise, state plan for documenting important protocol amendments | **N/A** |
| **Support:** | | | |
| Sources | 5a | Indicate sources of financial or other support for the review | **No financial resources to declare** |
| Sponsor | 5b | Provide name for the review funder and/or sponsor | **N/A** |
| Role of sponsor or funder | 5c | Describe roles of funder(s), sponsor(s), and/or institution(s), if any, in developing the protocol | **N/A** |
| INTRODUCTION | | |  |
| Rationale | 6 | Describe the rationale for the review in the context of what is already known | **Introduction section of the protocol.**  **(Pages 3-6)** |
| Objectives | 7 | Provide an explicit statement of the question(s) the review will address with reference to participants, interventions, comparators, and outcomes (PICO) | **Which bedside ultrasound techniques can clinicians utilise for risk assessment of AKI in hospitalised paediatric patients?**  **PECO framework (Table 1, page 8):**  **Population**: Paediatric patients (< 18 years of age)  **Exposure**: B-mode ultrasound, Doppler flow patterns and wavelength patterns (renal/ hepatic/ portal), right heart chamber measurements associated with AKI diagnosis  **Comparator**: none  **Outcome**: Acute kidney injury or renal dysfunction |
| METHODS | | | |
| Eligibility criteria | 8 | Specify the study characteristics (such as PICO, study design, setting, time frame) and report characteristics (such as years considered, language, publication status) to be used as criteria for eligibility for the review | Searches will be performed for English literature and unpublished, including grey literature available in the past 40 years. The inclusion and exclusion criteria are delineated in Table 2 (page 8). |
| Information sources | 9 | Describe all intended information sources (such as electronic databases, contact with study authors, trial registers or other grey literature sources) with planned dates of coverage | Literature search will include the following databases: MEDLINE (PubMed), Scopus, Cochrane Library, and CINHAL. In addition to the electronic database search, the reference lists of eligible articles will also be screened. |
| Search strategy | 10 | Present draft of search strategy to be used for at least one electronic database, including planned limits, such that it could be repeated | **Examples have been provided in table 3, pages 9 - 11.** |
| **Study records:** | | | |
| Data management | 11a | Describe the mechanism(s) that will be used to manage records and data throughout the review | The outputs of the search will be managed with the EndNote™ 20 [Endnote 20.6 (2001- 2020), Clarivate Analytics (US) LLC, Philadelphia, PA], management tool, and subsequently transferred to Covidence [Covidence systematic review software (2024), Veritas Health Innovation, Melbourne, Australia] for further study screening.  **(Step 3:page 8)** |
| Selection process | 11b | State the process that will be used for selecting studies (such as two independent reviewers) through each phase of the review (that is, screening, eligibility and inclusion in meta-analysis) | The inclusion of the identified studies, Abstract reviews, Full-text reviews, and Data Extraction will be carried out by two main reviewers (GL and NA); with the third (MK) and fourth reviewer (PMC) called in case of discordance.  **GL and NA will be working independently**. |
| Data collection process | 11c | Describe planned method of extracting data from reports (such as piloting forms, done independently, in duplicate), any processes for obtaining and confirming data from investigators | A data extraction template created by the authors during the Protocol Phase of the study will be uploaded into the Covidence Software and used independently by the primary reviewers: GL and NA for data extraction of the identified studies.  **(Data extraction template: Table 4: Pages 14 - 15)** |
| Data items | 12 | List and define all variables for which data will be sought (such as PICO items, funding sources), any pre-planned data assumptions and simplifications | **Table 4: pages 13 - 15**   - Study design (cohort/RCT/observational and also single/ multicentre) - Study period - Sample size and Paediatric population (neonate/ infants/child/teenager) - Inclusion and Exclusion criteria - Medical (ICU)/ Post Surgical patients - Ultrasound parameter used - Standard renal dysfunction diagnosis (KDIGO/ pRIFLE/ AKIN) also Serum Creatinine or Urine-Output or both |
| Outcomes and prioritization | 13 | List and define all outcomes for which data will be sought, including prioritization of main and additional outcomes, with rationale | 1. Acute kidney injury (Acute renal failure, Acute Kidney Injury, etc) by means of ultrasound diagnosis 2. Corroboration with serum creatinine/ urine output/ biomarkers 3. Sensitivity and Specificity of ultrasound technique 4. Mortalities |
| Risk of bias in individual studies | 14 | Describe anticipated methods for assessing risk of bias of individual studies, including whether this will be done at the outcome or study level, or both; state how this information will be used in data synthesis | Assessment of risk of bias of the included studies in this review paper will be performed using the Quality Assessment of comparative Diagnostic Accuracy Studies (QUADAS) -2 tool  **(Step 6, page 13)** |
| Data synthesis | 15a | Describe criteria under which study data will be quantitatively synthesised | Standardised mean differences and standard deviations for the different ultrasound parameters and diagnostic outcomes will be collated. |
|  | 15b | If data are appropriate for quantitative synthesis, describe planned summary measures, methods of handling data and methods of combining data from studies, including any planned exploration of consistency (such as I^2^, Kendall’s τ) | For the dichotomous variables (such AKI or No AKI), pooled risk ratio (RR) will be computed, with a 95% confidence interval (CI).  Additional to the CI, a p-value will be reported. However, for continuous variables, the unit of comparison will be mean differences with a 95% CI.  The chi-squared test and I^2^ will be used to evaluate the statistical  heterogeneity. Heterogeneity would be considered significant if I^2^  is greater than 40% (> 40%). In that event, a random effects model would be used for the pooled estimates, otherwise a fixed  effects model would be the reported. |
|  | 15c | Describe any proposed additional analyses (such as sensitivity or subgroup analyses, meta-regression) | Subgroup analysis would be explored where reported diagnostic parameter are reported for different organ such as where studies report on renal indices (renal resistive index, renal pulsative index, Renal Venous Stasis) vs cardiac or hepatic indices (Portal Pulsatility Fraction, Portal flow & Hepatic flow). |
|  | 15d | If quantitative synthesis is not appropriate, describe the type of summary planned | **N/A** |
| Meta-bias(es) | 16 | Specify any planned assessment of meta-bias(es) (such as publication bias across studies, selective reporting within studies) | Funnel plots would be performed to check the possible publication bias. |
| Confidence in cumulative evidence | 17 | Describe how the strength of the body of evidence will be assessed (such as GRADE) | The GRADE guidelines will be used to evaluate publication bias and selective reporting of included studies and be presented as plots of outcome variables against sample size.  **(Page 13)** |

*** It is strongly recommended that this checklist be read in conjunction with the PRISMA-P Explanation and Elaboration (cite when available) for important clarification on the items. Amendments to a review protocol should be tracked and dated. The copyright for PRISMA-P (including checklist) is held by the PRISMA-P Group and is distributed under a Creative Commons Attribution Licence 4.0.**

*From: Shamseer L, Moher D, Clarke M, Ghersi D, Liberati A, Petticrew M, Shekelle P, Stewart L, PRISMA-P Group. Preferred reporting items for systematic review and meta-analysis protocols (PRISMA-P) 2015: elaboration and explanation. BMJ. 2015 Jan 2;349(jan02 1):g7647.*

**APPENDIX B : QUADAS-2 (Risk of bias tool)**

**QUADAS-2**

**Phase 1: State the review question:**

| Patients (setting, intended use of index test, presentation, prior testing): |
| --- |
| Index test(s): |
| Reference standard and target condition: |

**Phase 2: Draw a flow diagram for the primary study**

|  |
| --- |

**Phase 3: Risk of bias and applicability judgments**

QUADAS-2 is structured so that 4 key domains are each rated in terms of the risk of bias and the concern regarding applicability to the research question (as defined above). Each key domain has a set of signaling questions to help reach the judgments regarding bias and applicability.

| \| Describe methods of patient selection: \| \| --- \|   **DOMAIN 1: PATIENT SELECTION**  **A. Risk of Bias**  Was a consecutive or random sample of patients enrolled? Yes/No/Unclear   Was a case-control design avoided? Yes/No/Unclear   Did the study avoid inappropriate exclusions? Yes/No/Unclear  **Could the selection of patients have introduced bias? RISK: LOW/HIGH/UNCLEAR**  **B. Concerns regarding applicability**   \| Describe included patients (prior testing, presentation, intended use of index test and setting): \| \| --- \|   Is there concern that the included patients do not match the review question? LOW/HIGH/UNCLEAR |
| --- | --- | --- |

| **DOMAIN 2: INDEX TEST(S)**  **If more than one index test was used, please complete for each test.**  **A. Risk of Bias**   \| **Describe the index test and how it was conducted and interpreted:** \| \| --- \|   - Were the index test results interpreted without knowledge of the results of the reference standard?  Yes/No/Unclear  - If a threshold was used, was it pre-specified? Yes/No/Unclear  **- Could the conduct or interpretation of the index test have introduced bias?**  **RISK: LOW/HIGH/UNCLEAR**  **B. Concerns regarding applicability**  - Is there concern that the index test, its conduct, or interpretation differ from the review question?  CONCERN: LOW/HIGH/UNCLEAR |
| --- | --- |

| **DOMAIN 3: REFERENCE STANDARD**  **A. Risk of Bias**   \| Describe the reference standard and how it was conducted and interpreted: \| \| --- \|   Is the reference standard likely to correctly classify the target condition?  Yes/No/Unclear  Were the reference standard results interpreted without knowledge of the results of the index test?  Yes/No/Unclear  **Could the reference standard, its conduct, or its interpretation have introduced bias?**  **RISK: LOW /HIGH/UNCLEAR**  **B. Concerns regarding applicability**  Is there concern that the target condition as defined by the reference standard does not match the review question?  CONCERN: LOW /HIGH/UNCLEAR |
| --- | --- |

| **DOMAIN 4: FLOW AND TIMING**  **A. Risk of Bias**   \| Describe any patients who did not receive the index test(s) and/or reference standard or who were excluded from the 2x2 table (refer to flow diagram):  Describe the time interval and any interventions between index test(s) and reference standard: \| \| --- \|   Was there an appropriate interval between index test(s) and reference standard?  Yes/No/Unclear   Did all patients receive a reference standard? Yes/No/Unclear   Did patients receive the same reference standard? Yes/No/Unclear   Were all patients included in the analysis? Yes/No/Unclear  **Could the patient flow have introduced bias? RISK: LOW /HIGH/UNCLEAR** |
| --- | --- |
